# Finite element analysis of the mechanical behavior of stiffened cold-formed built-up CFST flat columns

**DOI:** 10.1371/journal.pone.0361413

**Published:** 2026-10-28

**Authors:** Wenjing Yu, Haijie Wang, Qing Jiang, Hanqin Wang

**Affiliations:** 1 Anhui Technical College of Water Resources and Hydroelectric Power, Hefei, China; 2 School of Civil Engineering, Hefei University of Technology, Hefei, China; 3 School of Architecture and Art, Hefei University of Technology, Hefei, China; Universiti Teknologi Malaysia, MALAYSIA

## Abstract

To address insufficient lateral restraint and the susceptibility of the flat walls of traditional concrete-filled steel tubular (CFST) rectangular flat columns, a built-up rectangular CFST flat column with integrated longitudinal cold-formed stiffening ribs is proposed. The FE model was validated against the global response of a welded-plate CFST flat-column specimen. The model was then used to study the axial-compression and quasi-static cyclic behavior of the proposed columns. The coupled effects of aspect ratio, tube thickness, and rib dimensions were evaluated. The individual effects of tube thickness and rib dimensions under axial compression were further examined through the supplementary decoupled analysis. The results indicate that, compared with the non-stiffened control specimen, the stiffened configuration provides higher axial and cyclic load-bearing capacity, and more effective suppression of local outward bulging. Within the investigated parameter range, the coupled increase in tube thickness and rib dimensions improves axial and cyclic performance, while the decoupled analysis indicates that tube thickness is the dominant parameter governing axial load-bearing capacity and rib dimensions mainly contribute to local outward deformation control and post-peak load retention. These findings provide a reference for the parametric design and engineering application of stiffened cold-formed built-up rectangular flat CFST columns.

## 1. Introduction

Driven by the goals of carbon peaking and carbon neutrality, green construction and building industrialization have been continuously promoted. Steel structures, featuring low self-weight, high strength, a high degree of prefabrication, and favorable seismic performance, have gradually become a key direction for high-quality development of industrialized construction [1,2]. Concrete-filled steel tubular (CFST) columns, as critical load-bearing members, have been widely used in buildings and bridges due to the composite action between steel tubes and concrete, as well as their durability and ductility advantages [3].

In prefabricated residential buildings, the long-standing issue of “exposed columns” constrains architectural aesthetics and space utilization. Compared with circular sections, rectangular sections better match wall thickness and facilitate concealing columns within walls, thereby improving layout efficiency and visual appearance. When the ratio of the long side to the short side of a rectangular section is relatively large, the member is commonly referred to as a CFST flat column. Such members must maintain adequate load capacity and ductility while meeting the requirement of concealed placement. Owing to the pronounced difference between the long and short sides, the stress state and restraint conditions of the plate elements are highly non-uniform, making the member more sensitive to local buckling and strength degradation. Research on this configuration remains relatively limited.

From a manufacturing perspective, CFST steel tubes are mainly produced by two methods: hot-rolled plate welding and cold-forming [4,5]. Cold-forming enables a section to be fabricated by bending steel plates and then assembling/welding them, resulting in fewer welds, higher production efficiency, improved dimensional accuracy, and shorter construction cycles. This technique therefore has potential for rectangular flat-column applications. However, cold-forming introduces corner radii, strain hardening, residual stresses, and initial geometric imperfections. Together with weld locations and fabrication tolerances, these forming effects may reduce the local stability of thin plates and influence steel–concrete composite action. To restrain local buckling of thin flat plates and improve confinement and ductility, installing longitudinal/transverse stiffening ribs inside steel tubes is considered an effective detailing measure.

Existing studies have mainly verified the beneficial role of ribs for thin-walled CFST members, with emphasis on square sections through experimental and numerical investigations. Zhang et al. [6,7] reported that bidirectional ribs significantly enhance axial capacity and energy dissipation based on axial-compression and seismic tests. Xu et al. [8] showed that ribs can change the buckling mode and force-transfer path in stub-column tests. Wang et al. [9] compared the effects of material strength, axial load ratio, width-to-thickness ratio, and slenderness ratio and proposed a simplified hysteresis model. Zheng et al. [10] and Ma et al. [11] verified that ribs can delay local buckling and increase capacity under different rib forms and material conditions. More recently, Hassanein et al. [12] studied square cold-formed concrete-filled double-skin stiffened slender columns and further emphasized the importance of width-to-thickness ratio and slenderness effects. In general, prior work has focused on square sections or rectangular sections with relatively small aspect ratios. For cold-formed thin-walled CFST flat columns with significantly increased aspect ratios, the mechanisms of local buckling control, the effectiveness of rib layouts in mitigating non-uniform confinement, and systematic evaluation of axial and quasi-static cyclic behavior require further investigation.

Meanwhile, previous experimental studies on rectangular CFST flat columns assembled from hot-rolled angle or channel sections and internal steel plates [13] have clarified their seismic behavior, failure characteristics, and skeleton-curve features, providing relevant experimental understanding for the numerical investigation of improved flat-column configurations. Accordingly, this study proposes a stiffened cold-formed built-up rectangular CFST flat-column configuration and designs 10 original specimens, including nine stiffened specimens and one non-stiffened counterpart. FE analyses are conducted in ABAQUS under axial compression and quasi-static cyclic loading. The rounded corners and inward-folded lips forming the longitudinal stiffening ribs are explicitly represented in the FE geometry, whereas manufacturing-induced strain hardening, residual stresses, and initial geometric imperfections are not considered separately. To further distinguish the individual effects of tube thickness and rib dimensions, six supplementary axial-compression models are established for decoupled parametric analysis. The results are expected to provide a reference for the design and application of stiffened cold-formed built-up CFST flat columns in prefabricated residential buildings.

## 2. Flat-column configuration

The proposed stiffened CFST flat column is assembled from four cold-formed lipped channel plates. Each plate consists of an outer wall segment, a rounded corner, a side wall segment, and an inward lip formed during cold bending. The four plates are longitudinally butt-welded at the midpoints of the four sides to form a closed rectangular steel tube. After assembly, the inward lips of the four plates act as internal longitudinal stiffening ribs. Therefore, the ribs are integrated with the cold-formed plates rather than being separately welded to the tube wall. Concrete is then filled into the steel tube to form the composite member. Loading plates are welded at both ends to satisfy the axial-compression and quasi-static cyclic loading requirements. Fig 1 shows the schematic of the stiffened CFST flat column.

**Fig 1:**
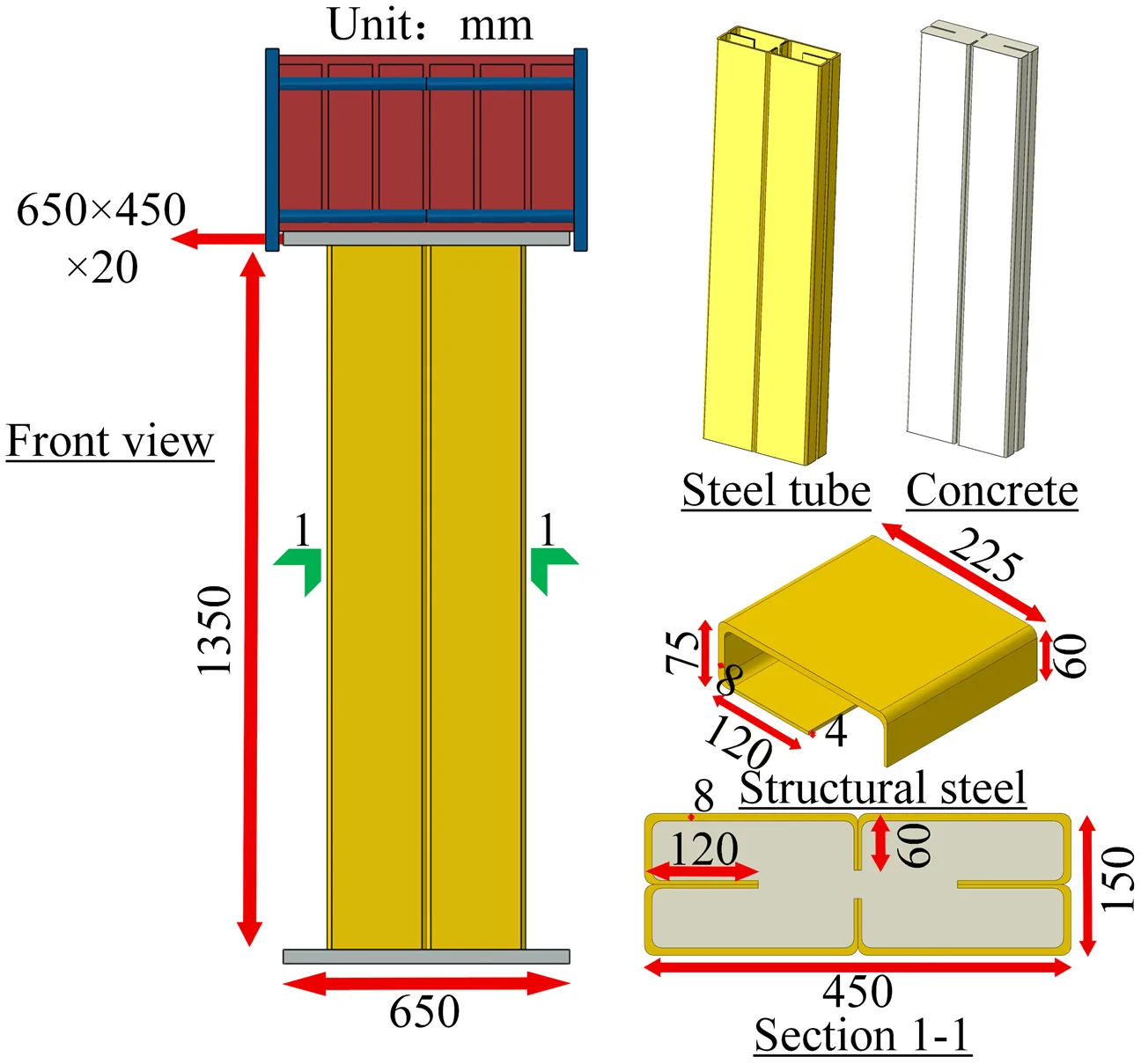
Schematic of the stiffened CFST flat column.

A total of 10 original specimens were designed, as listed in Table 1. The specimen ID follows the form Cα-Tt-Rr, where α denotes the section aspect ratio, t denotes the steel-tube thickness in millimeters, and r denotes the rib-dimension group. For example, C2.5-T4-R80 represents a specimen with a section aspect ratio of 2.5, a tube thickness of 4 mm, and rib dimensions of 80/40 mm (short side/long side). The non-stiffened control specimen is denoted as C2-T2-R0, which has the same outer section size and tube thickness as C2-T2-R40 but without internal ribs. The original specimen matrix includes three section aspect ratios of 2.0, 2.5, and 3.0, three tube thicknesses of 2, 4, and 8 mm, and three corresponding rib-dimension groups of 40/20, 80/40, and 120/60 mm. For the axial-compression specimens, the member height was taken as three times the long side of the cross-section; therefore, the heights of the specimens with section sizes of 300 × 150, 375 × 150, and 450 × 150 mm were 900, 1125, and 1350 mm, respectively. For the quasi-static cyclic specimens, the member height was uniformly set to 1600 mm. In the original matrix, the tube thickness and rib dimensions were increased simultaneously to represent practical strengthening levels of the built-up cold-formed section. Therefore, the results obtained from the original specimens mainly reflect the coupled effect of tube thickness and rib dimensions.

**Table 1:** Design of stiffened cold-formed built-up CFST flat-column specimens.

Specimen ID	Section size (mm)	Aspect ratio	Tube thickness (mm)	Rib dimension (mm)	
				short side	long side
C2-T2-R0	300 × 150	2	2	None	
C2-T2-R40	300 × 150	2	2	40	20
C2-T4-R80			4	80	40
C2-T8-R120			8	120	60
C2.5-T2-R40	375 × 150	2.5	2	40	20
C2.5-T4-R80			4	80	40
C2.5-T8-R120			8	120	60
C3-T2-R40	450 × 150	3	2	40	20
C3-T4-R80			4	80	40
C3-T8-R120			8	120	60

To further distinguish the individual effects of tube thickness and rib dimensions, six supplementary axial-compression models were established based on the representative section with an aspect ratio of 2.5. Together with the three original specimens C2.5-T2-R40, C2.5-T4-R80, and C2.5-T8-R120, these supplementary models form a 3 × 3 parameter matrix with tube thicknesses of 2, 4, and 8 mm and rib-dimension groups of 40/20, 80/40, and 120/60 mm. The six supplementary models are C2.5-T2-R80, C2.5-T2-R120, C2.5-T4-R40, C2.5-T4-R120, C2.5-T8-R40, and C2.5-T8-R80. These models were used only for the axial-compression decoupled analysis. Their member height was 1125 mm, consistent with the height rule adopted for the original axial-compression specimen with a 375 × 150 mm section. The quasi-static cyclic analysis was still conducted using the original 10 specimens under the same loading protocol to maintain consistent comparison among the cyclic models.

## 3. Finite element analysis of axial compression behavior

### 3.1 Finite element model

A three-dimensional solid FE model for axial compression of stiffened cold-formed built-up CFST flat columns was established in ABAQUS. The axial-compression FE analysis included the 10 original specimens and the 6 supplementary decoupled specimens described in Section 2. Loading plates were set at both ends and modeled as linear elastic material with an elastic modulus of 2 × 10^5^ MPa. The loading plates, concrete, and steel tube were all modeled using C3D8R solid elements. For modeling simplicity, welds were neglected.

The steel–concrete interaction was modeled using surface-to-surface contact, with the steel tube defined as the master surface and the core concrete as the slave surface. Hard contact was adopted in the normal direction, and Coulomb friction was used in the tangential direction with a friction coefficient of 0.25.

The steel tube was discretized using two solid-element layers across the thickness, and the meshes at the rounded corners, rib-folded regions, and steel-concrete contact interfaces were refined. The refinement was adopted to better capture local stress concentration, contact interaction, and outward deformation of the flat steel plates. Fig 2 shows the finite element model of the stiffened CFST flat column under axial compression.

**Fig 2:**
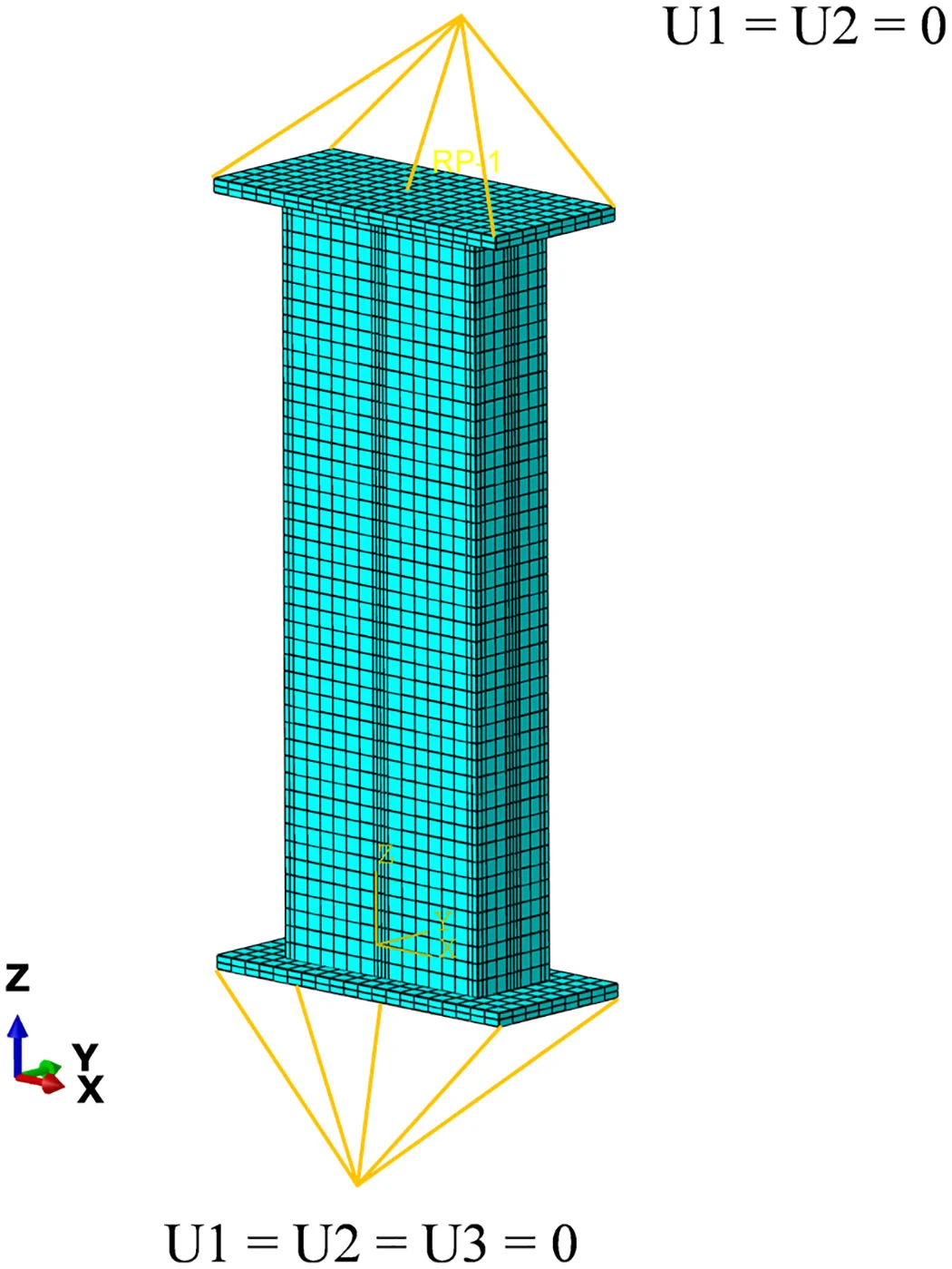
Finite element model for axial compression.

A reference point, RP-1, was created at the top of the FE model and coupled with the loading plate. A kinematic coupling constraint was adopted, coupling the three translational degrees of freedom between RP-1 and the loading plate while releasing the rotational degrees of freedom.

The boundary conditions were set as follows: the translational degrees of freedom in the X and Y directions of RP-1 were constrained (the Z direction was released to allow axial loading); the bottom surface of the base plate was constrained in all three translational degrees of freedom, with rotational degrees of freedom released.

A sensitivity analysis was performed using model C2.5-T4-R80. Three mesh schemes were considered: baseline, refined, and coarse. The friction coefficient was set to 0.10, 0.20, 0.25, 0.30, and 0.60. The load–displacement curves were nearly identical. Compared with the baseline model, the maximum differences in ultimate axial capacity were 0.28% for mesh and 0.24% for friction. Thus, mesh and friction had little effect on the axial response within the ranges studied.

### 3.2 Constitutive models

In the axial-compression analysis, the steel tube was modeled using a bilinear elastic-plastic constitutive model with isotropic hardening to represent the post-yield hardening behavior of Q235 steel. The yield strength was taken as 235 MPa, and the tangent modulus of the post-yield hardening branch was determined according to the slope corresponding to a plastic strain of 0.05. For concrete, the built-in Concrete Damaged Plasticity (CDP) model in ABAQUS was adopted, the compressive stress-strain relationship of the core concrete was defined according to Han’s confined concrete model, while the CDP framework was used to describe the nonlinear damage behavior of concrete in the FE analysis. The physical meanings and values of the CDP parameters are listed in Table 2.

**Table 2:** CDP model parameter setting.

*Φ*	*ε*	*f*_b0_/*f*_c0_	*K*_c_	*μ*	*E*_c_
30°	0.1	1.16	0.667	0.0005	$4700\sqrt{f_{\text{c}}^{{\;}^{\prime }}}$

In Table 2, Φ is the dilation angle; ε is the eccentricity; *f*_b0_ and *f*_c0_ are the initial biaxial and uniaxial compressive strengths of concrete, respectively; *K*_c_ is the ratio of the second stress invariant on the tensile meridian to that on the compressive meridian; *μ* is the viscosity parameter; *E*_c_ is the elastic modulus of concrete.

The compressive stress–strain relationship of concrete followed the confined concrete constitutive model proposed by Han [14]. Based on extensive test data and considering the confinement-effect coefficient of CFST, Han proposed a stress–strain model for concrete in the core region of CFST members. Eqs. (1)–(7) describe the compressive stress–strain relationship of confined concrete.


$$\begin{array}{c} y=\left\{ \begin{array}{c} \begin{array}{cc} 2x-x^{\text{2}} & \;x\leq 1 \end{array}\\ \begin{array}{cc} \frac{x}{\beta_{\text{0}}{\left(x-1\right)}^{\eta }+x} & x>1 \end{array} \end{array}\right. \end{array}$$ (1)

$$\begin{array}{c} \sigma =\left[1+\left(-0.0135\xi^{\text{2}}+0.1\xi \right){\left(\frac{24}{f_{c}{\;}^{\prime }}\right)}^{0.45}\right]\cdot f_{c}{\;}^{\prime } \end{array}$$ (2)

$$\varepsilon_{\text{0}}=\varepsilon_{\text{cc}}+\left[1330+760\left(\frac{f_{c}^{'}}{24}-1\right)\right]\zeta^{0.2}$$ (3)

$$\begin{array}{c} \varepsilon_{\text{cc}}=1300+12.5f_{\text{c}}^{\prime } \end{array}$$ (4)

$$\begin{array}{c} \eta =1.6+1.5/x \end{array}$$ (5)

$$\begin{array}{c} \beta =\left\{ \begin{array}{c} \begin{array}{cc} \frac{{f_{\text{c}}^{{\;}^{\prime }}}^{0.1}}{1.35\sqrt{1+\xi }} & \xi \leq 3.0 \end{array}\\ \begin{array}{cc} \frac{{f_{\text{c}}^{{\;}^{\prime }}}^{0.1}}{1.35\sqrt{1+\xi }\cdot {\left(\xi -2\right)}^{\text{2}}} & \xi >3.0 \end{array} \end{array}\right. \end{array}$$ (6)

$$\begin{array}{c} \xi =\frac{A_{\text{s}}f_{y}}{A_{\text{c}}f_{ck}} \end{array}$$ (7)

where *ξ* is the confinement-effect coefficient; *η* is a modification coefficient; As is the cross-sectional area of the steel tube; *A*_c_ is the cross-sectional area of concrete; *f*_y_ is the yield strength of the steel tube; *f*_ck_ is the standard value of axial compressive strength of concrete.

Han’s confined concrete model has been widely used to describe the compressive stress-strain behavior of core concrete in CFST members. In this study, this model was adopted as an equivalent constitutive model for the core concrete. Previous experimental studies on rectangular CFST flat columns [13], together with the validation result in Section 3.3, provide a basis for applying the adopted FE modeling approach to the analysis of flat-column behavior. To avoid overestimating the confinement effect in the proposed stiffened section, the rib area was not included in the calculation of the confinement coefficient.

The tensile stress–strain relationship of concrete adopted the model provided in the Chinese code for concrete structures [15], Eqs. (8)–(11) describe this relationship.


$$\begin{array}{c} \sigma =\left(1-d_{\text{t}}\right)E_{\text{c}}\varepsilon \end{array}$$ (8)

$$\begin{array}{c} d_{\text{t}}=\left\{ \begin{array}{c} \text{1}-\rho_{\text{t}}\left[\text{1.2}-\text{0.2}x^{\text{2}}\right]\;x\leq \text{1}\\ 1-\frac{\rho_{\text{t}}}{\alpha_{\text{t}}{\left(x-\text{1}\right)}^{\text{1.7}}+x}\;x>\text{1} \end{array}\right. \end{array}$$ (9)

$$\begin{array}{c} x=\frac{\varepsilon }{\varepsilon_{\text{t}}} \end{array}$$ (10)

$$\begin{array}{c} \rho_{\text{t}}=\frac{f_{\text{t}}}{E_{\text{c}}\varepsilon_{\text{t}}} \end{array}$$ (11)

where *σ* is the tensile stress of concrete; *α*_t_ is the reference value for the descending branch.

To facilitate subsequent experimental verification, the concrete strength in the FE models was set to C30 and the steel tube material to Q235; namely, *f*_cu,k_ = 30 N/mm^2^ and *f*_y_ = 235 N/mm^2^ were used throughout.

### 3.3 Model Validation

To validate the FE modeling approach, an FE model was developed for the CFST flat-column specimen reported in Ref [16]. This specimen was fabricated by welding flat steel plates rather than by cold-forming. Fig 3 compares the simulated and experimental load–displacement curves, which show good agreement and confirm that the adopted approach can reasonably reproduce the global response of a high-aspect-ratio CFST flat column. The specimens in Ref [13]. were assembled from hot-rolled angle or channel sections and internal steel plates. Therefore, neither reference specimen had the cold-formed lipped built-up configuration proposed in this study. Accordingly, the validation applies primarily to the global member response. Although the rounded corners and inward-folded lips forming the longitudinal stiffening ribs are represented in the FE geometry of the proposed specimens, manufacturing-induced strain hardening, residual stresses, and initial geometric imperfections were not independently validated.

**Fig 3:**
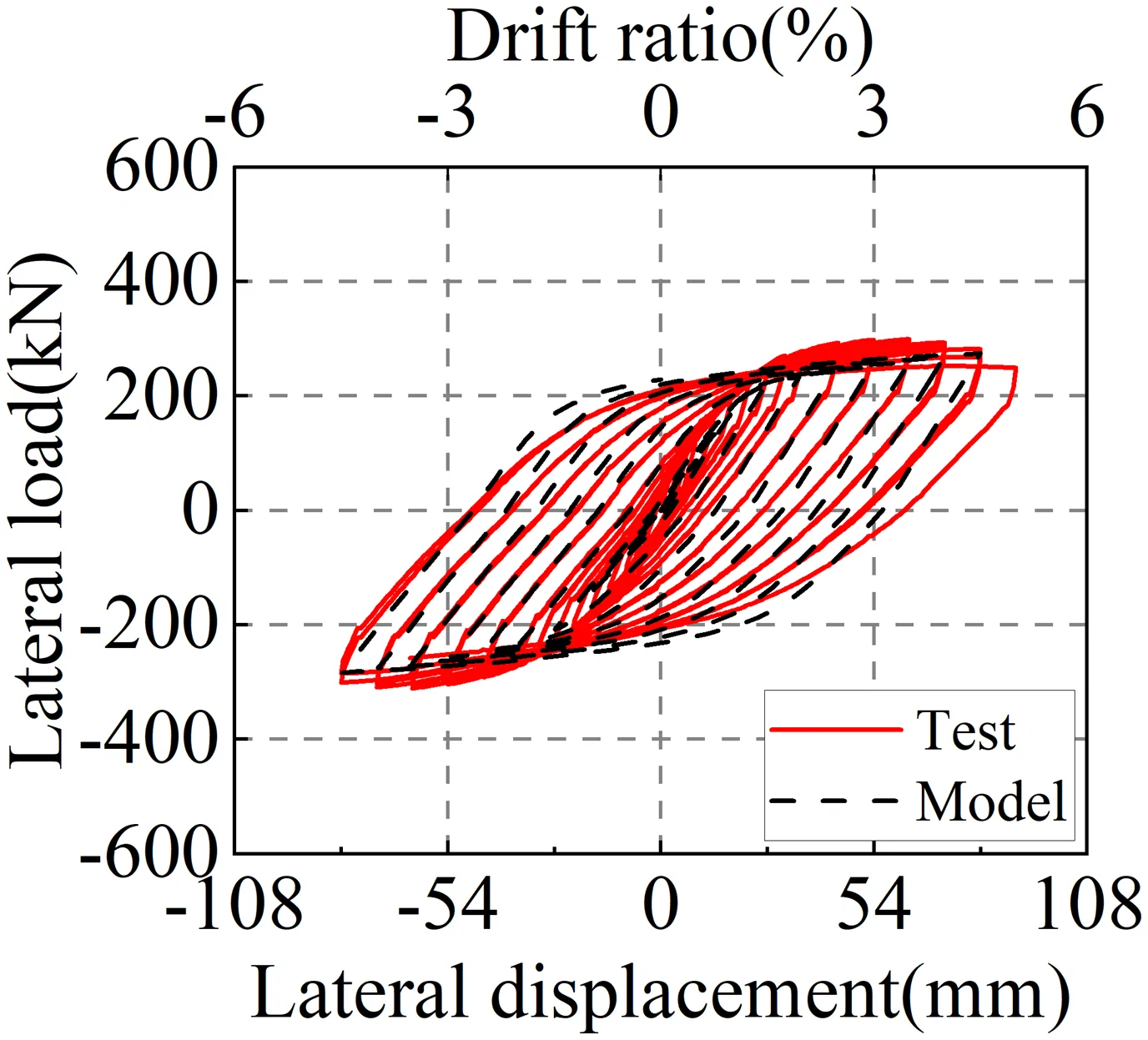
Comparison between simulation and test results in Ref. [16].

### 3.4 Simulation results

This section discusses the axial-compression results of the 10 original specimens, in which the tube thickness and rib dimensions were varied simultaneously according to the original strengthening levels. Fig 4 shows the von Mises equivalent stress distributions of the steel tube for the axial-compression specimens. Fig 5 shows the corresponding stress distributions of the concrete.

**Fig 4:**
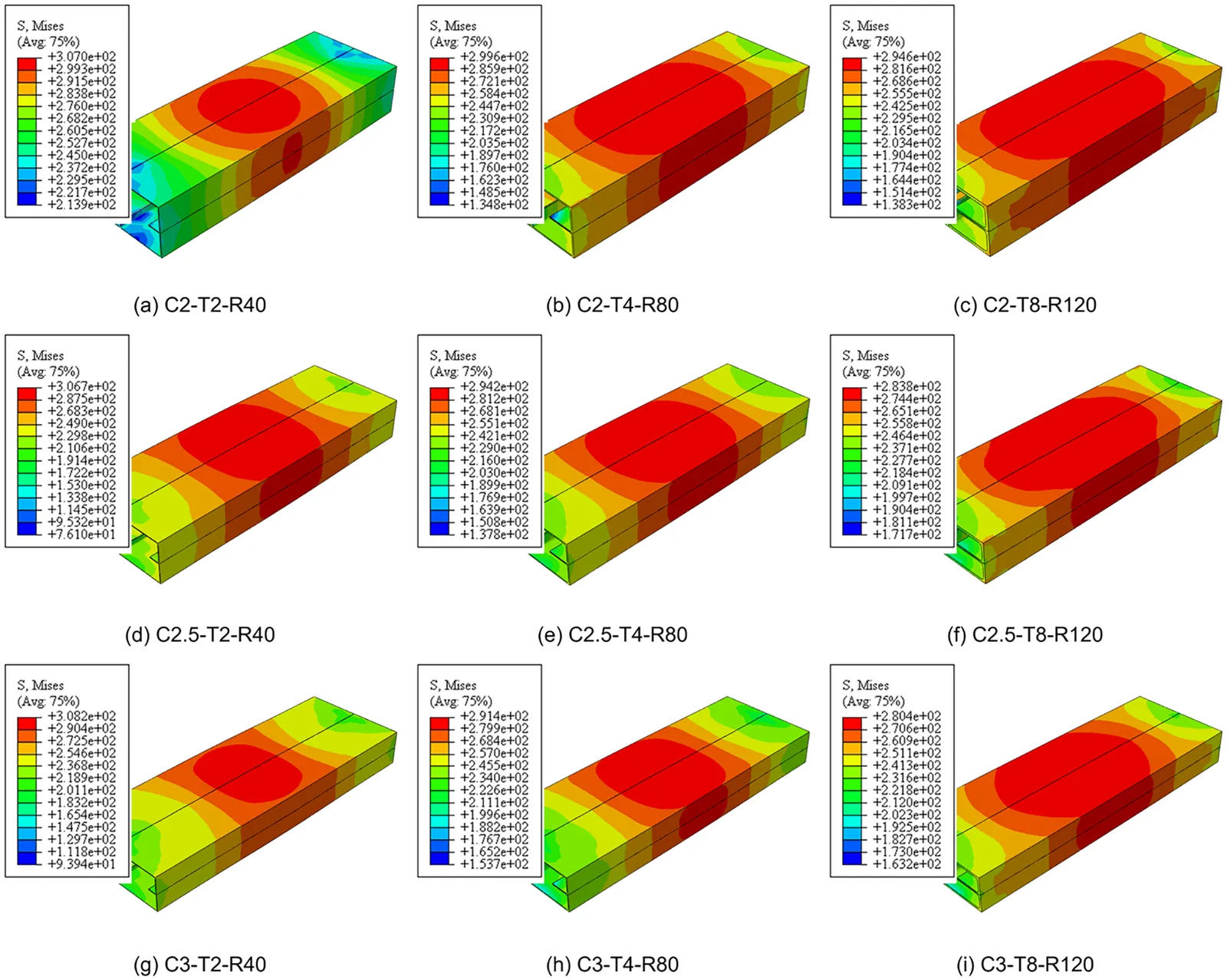
Steel-tube stress distributions of axial-compression specimens.

**Fig 5:**
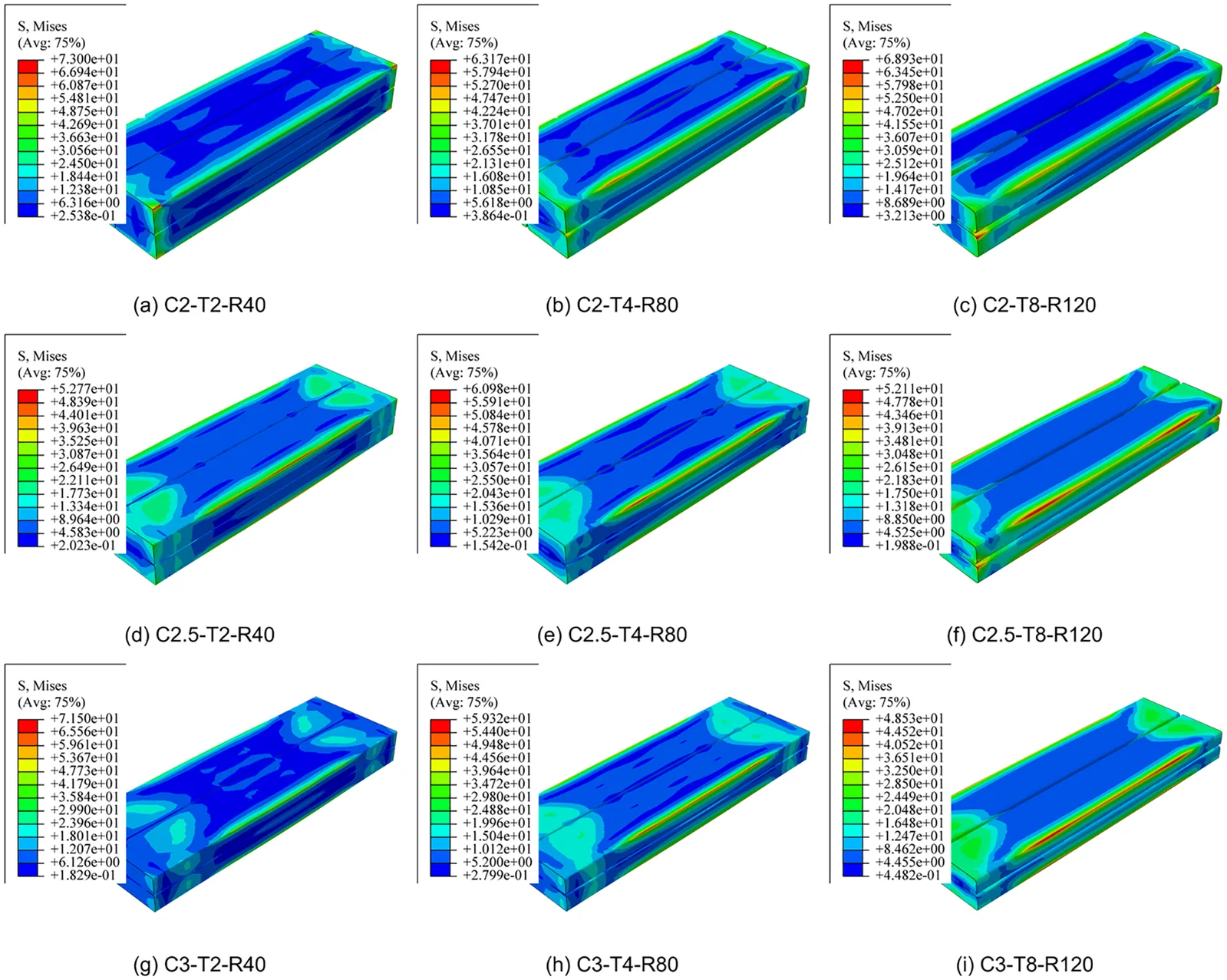
Concrete stress distributions of axial-compression specimens.

As shown in Figs 4–5, all nine stiffened flat columns reached the yield stress during loading, and the equivalent stress in the steel tube was more concentrated near the mid-height section. With the simultaneous increase in tube thickness and rib dimensions, the steel area, plate out-of-plane stiffness, and confinement effect on the core concrete increased, which contributed to the improvement in axial load-bearing capacity and the restraint of local outward deformation.

Fig 6 shows the load–displacement curves of the original specimens with different tube thicknesses and corresponding rib dimensions. Fig 7 shows the load–displacement curves of flat columns with different aspect ratios. Fig 8 compares the load–displacement curves of the non-stiffened and stiffened specimens with the same outer section size and tube thickness.

**Fig 6:**
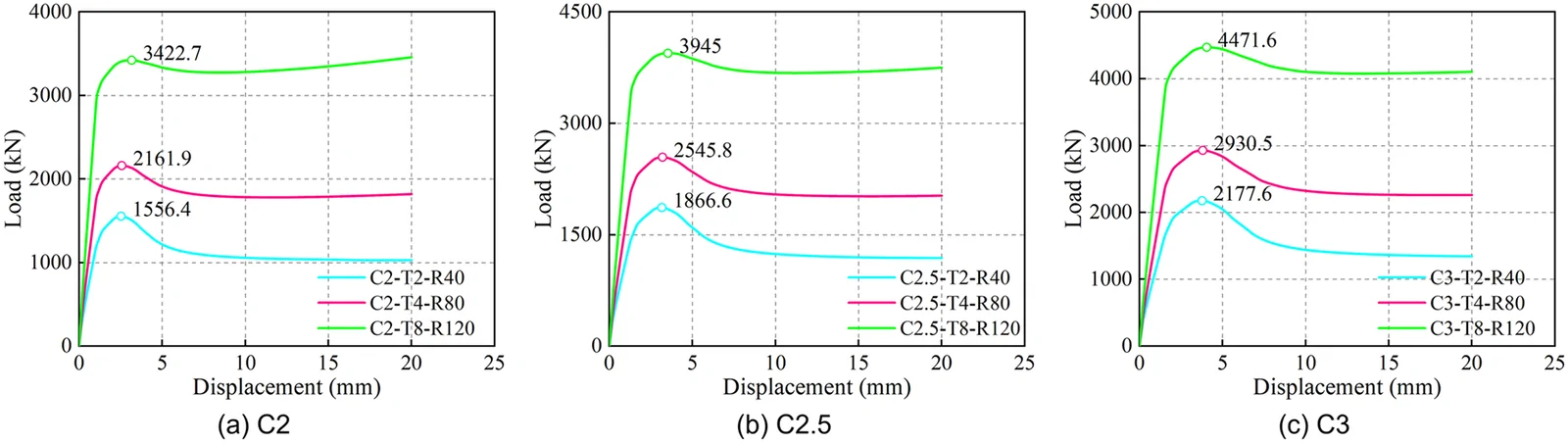
Load–displacement curves of flat columns with different tube thicknesses and corresponding rib dimensions.

**Fig 7:**
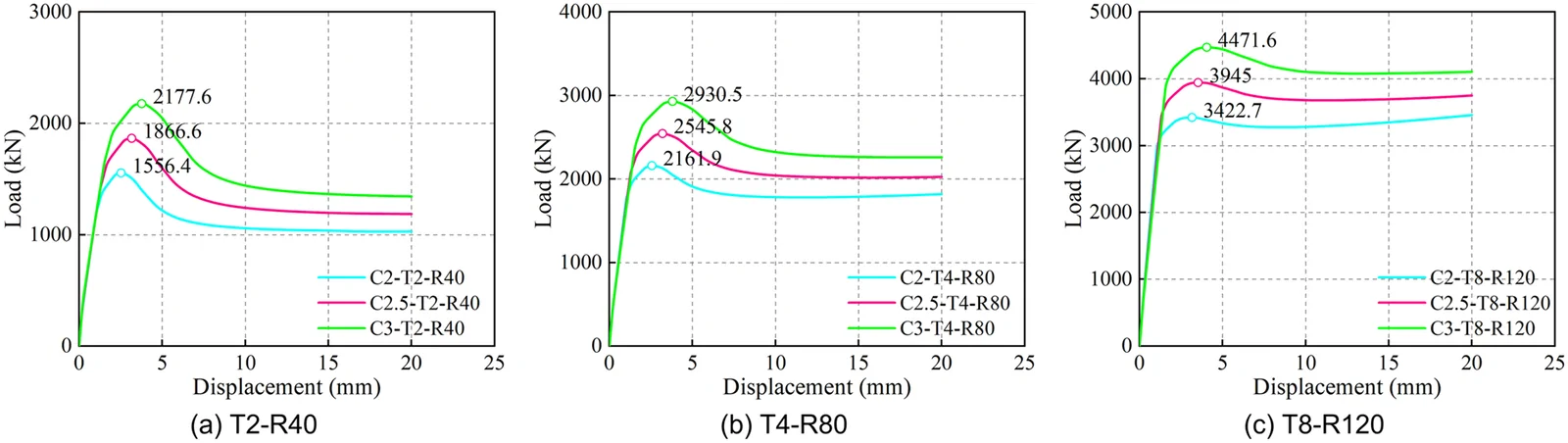
Load–displacement curves of flat columns with different aspect ratios.

**Fig 8:**
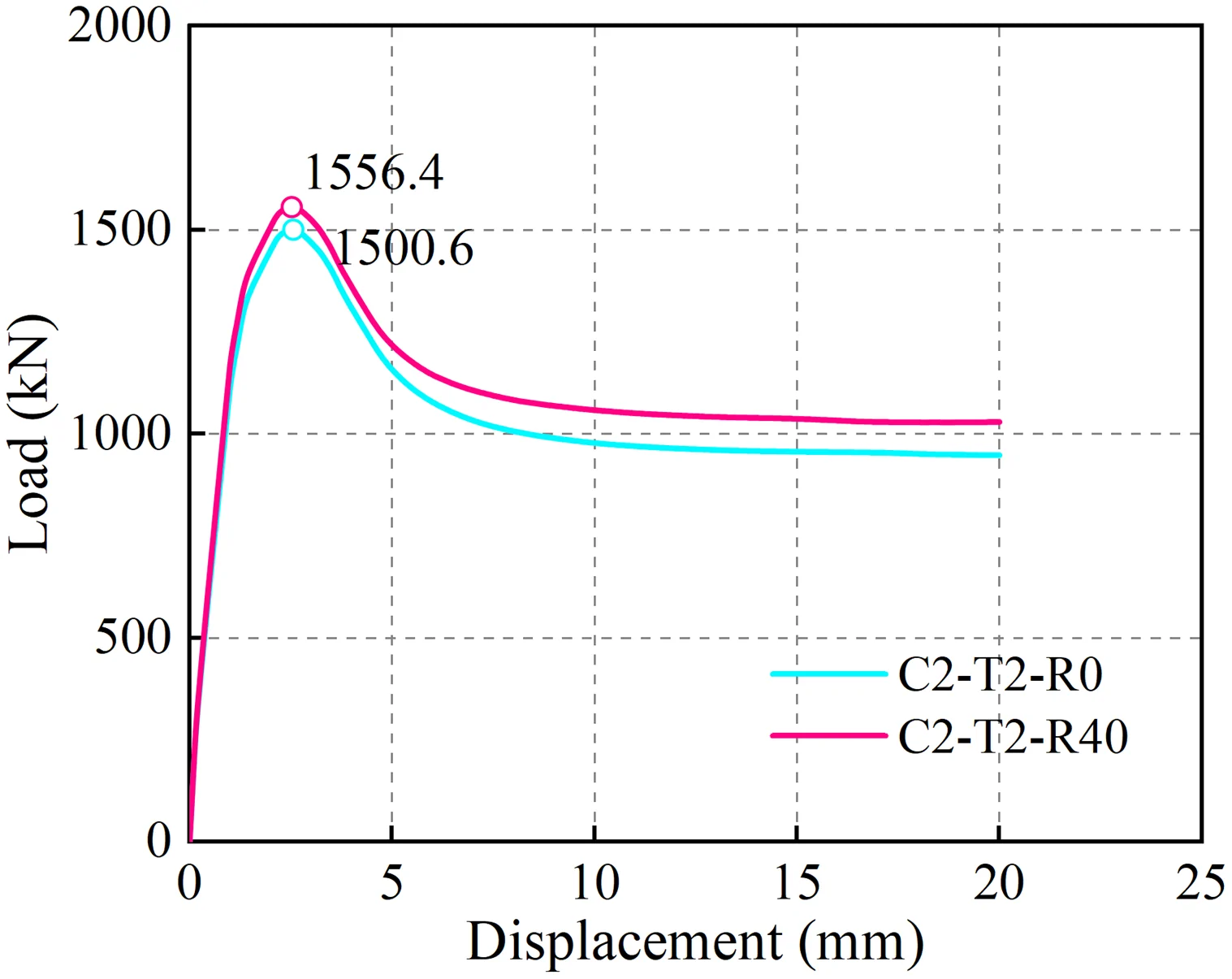
Load–displacement curves of the non-stiffened and stiffened specimens.

As shown in Figs 6-7, all specimens exhibit similar load-displacement response trends. In the initial stage, the load increases approximately linearly with displacement. After yielding, the response enters an elastic-plastic stage and gradually reaches the peak load, followed by a descending branch and a relatively stable residual load stage. For specimens with the same section aspect ratio, the axial load-bearing capacity increases markedly with the simultaneous increase in tube thickness and rib dimensions from T2-R40 to T8-R120. The peak load increases by 119.9%, 111.3%, and 105.4% for the C2, C2.5, and C3 groups, respectively. This improvement can be attributed to the increased steel area, reduced plate width-to-thickness ratio, and enhanced confinement effect on the core concrete. For specimens with the same coupled thickness-rib group, increasing the section aspect ratio from 2.0 to 3.0 also increases the peak load, but the increase is relatively smaller, ranging from 30.6% to 39.9%. Meanwhile, the displacement corresponding to the peak load increases with the section aspect ratio, indicating improved deformation capacity within the investigated range. Because tube thickness and rib dimensions are coupled in the original specimen matrix, their individual effects are further examined in the supplementary decoupled analysis.

Fig 8 compares the load-displacement curves of the non-stiffened specimen C2-T2-R0 and the stiffened specimen C2-T2-R40. The two specimens have the same outer section size and tube thickness, and the only difference is the presence of internal longitudinal ribs. Compared with C2-T2-R0, the peak load of C2-T2-R40 increases from 1500.6 kN to 1556.4 kN, corresponding to an increase of 3.7%. In addition, the stiffened specimen maintains a slightly higher load level after the peak, indicating that the internal ribs contribute to local restraint and residual load retention under axial compression.

### 3.5 Decoupled analysis of tube thickness and rib dimensions

In the original specimen matrix, the tube thickness and rib dimensions were varied simultaneously. Specifically, when the tube thickness increased from 2 mm to 4 mm and 8 mm, the rib dimensions also increased from 40/20 mm to 80/40 mm and 120/60 mm. This design represents practical strengthening levels in engineering applications, but it also couples the effects of the two parameters. Therefore, a supplementary decoupled parametric analysis was conducted to distinguish the individual effects of tube thickness and rib dimensions.

The decoupled analysis was performed using the section with an aspect ratio of 2.5, corresponding to a section size of 375 × 150 mm. Three tube thicknesses of 2, 4, and 8 mm and three rib-dimension groups of 40/20, 80/40, and 120/60 mm were combined to form a 3 × 3 parameter matrix. Among these models, C2.5-T2-R40, C2.5-T4-R80, and C2.5-T8-R120 were already included in the original specimen matrix, while the other six models were newly added for the axial-compression decoupled analysis. This matrix enables the effect of tube thickness to be examined at a fixed rib dimension and the effect of rib dimensions to be examined at a fixed tube thickness.

Fig 9 shows the load-displacement curves of the decoupled specimens with fixed rib dimensions and different tube thicknesses. For all three rib-dimension groups, increasing the tube thickness from 2 mm to 4 mm and 8 mm markedly increases the axial load-bearing capacity. Taking the R40 group as an example, the peak load increases by 30.7% and 88.6% when the tube thickness increases from 2 mm to 4 mm and 8 mm, respectively. Similar trends are observed for the R80 and R120 groups. This indicates that tube thickness is the dominant parameter governing axial load-bearing capacity, mainly because it increases the steel area, reduces the plate width-to-thickness ratio, and enhances the confinement effect on the core concrete.

**Fig 9:**
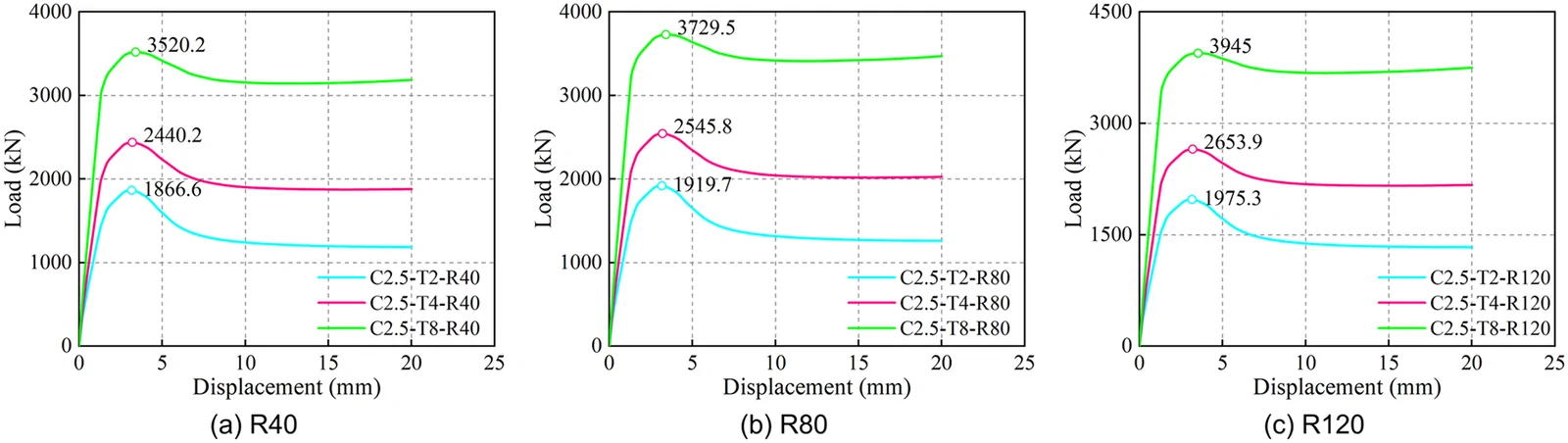
Load-displacement curves of decoupled specimens with fixed rib dimensions and different tube thicknesses.

Fig 10 shows the load-displacement curves of the decoupled specimens with fixed tube thickness and different rib dimensions. Compared with tube thickness, the effect of rib dimensions on peak load is more moderate. When the tube thickness is fixed at 2, 4, and 8 mm, increasing the rib dimensions from R40 to R120 increases the peak load by 5.8%, 8.8%, and 12.1%, respectively. These results indicate that rib enlargement provides a positive but secondary contribution to axial resistance.

**Fig 10:**
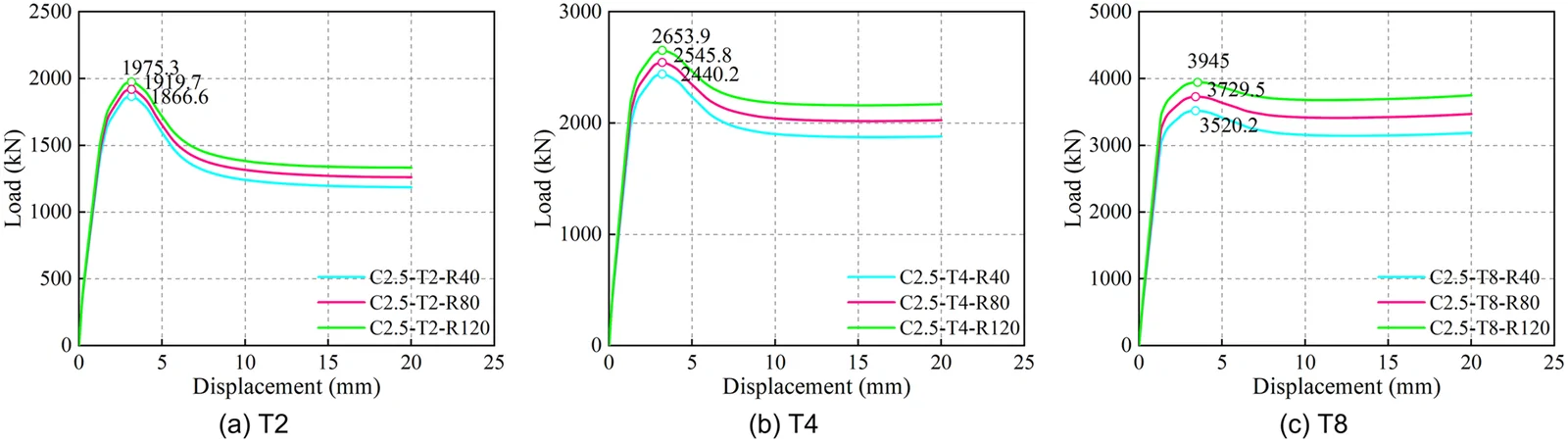
Load-displacement curves of decoupled specimens with fixed tube thickness and different rib dimensions.

Fig 11 compares the U1 displacement distributions of specimens with different rib dimensions at the same axial displacement. The three specimens have the same section aspect ratio and tube thickness, and only the rib dimensions are varied. As the rib dimensions increase from R40 to R120, the maximum lateral outward displacement decreases by approximately 6.9%. This indicates that increasing rib dimensions provides additional local restraint to the flat steel plate and moderately suppresses outward bulging.

**Fig 11:**
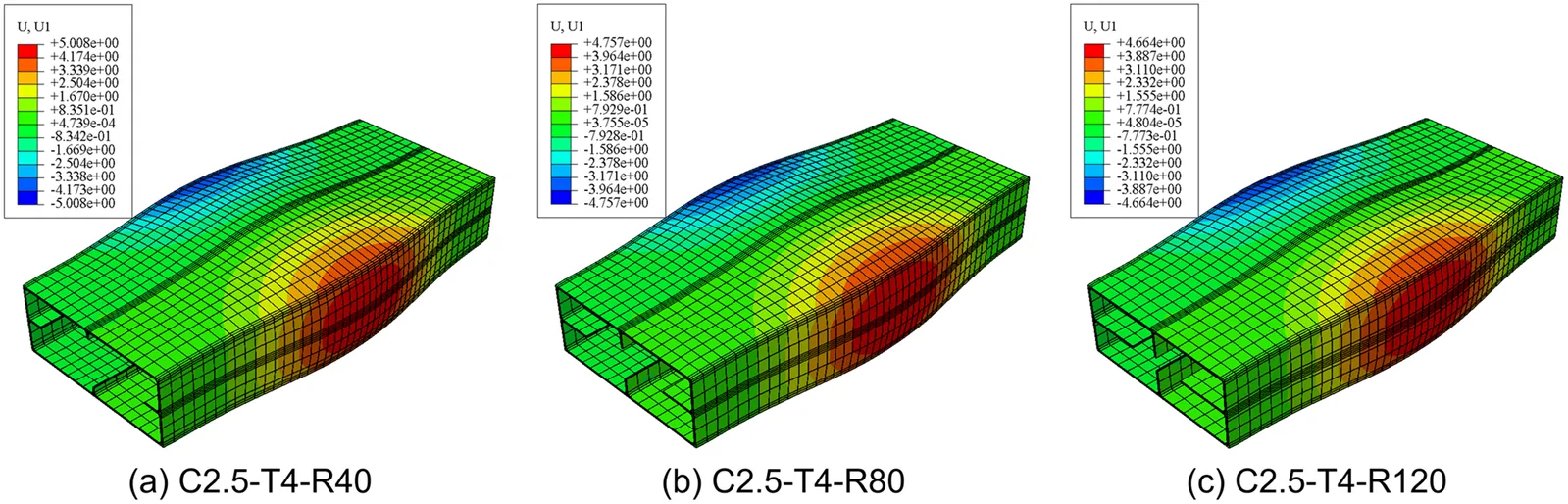
U1 displacement distributions of specimens with different rib dimensions at an axial displacement of 20 mm.

Overall, the decoupled analysis shows that the axial-compression improvement observed in the original coupled specimens is mainly governed by the increase in tube thickness. Rib dimensions provide an auxiliary contribution, mainly reflected in local deformation control and post-peak load retention. Therefore, the original coupled thickness-rib design should be interpreted as a practical strengthening scheme rather than evidence of the isolated effect of rib dimensions.

## 4. Finite element analysis of seismic behavior

### 4.1 Finite element model

A three-dimensional solid FE model for seismic behavior of stiffened cold-formed built-up CFST flat columns was established in ABAQUS. A loading device was modeled above the loading plate to match the experimental boundary conditions. The contact type, friction coefficient, and meshing strategy for concrete–steel interaction were the same as those in the axial-compression model. Fig 12 shows the finite element model for cyclic analysis.

**Fig 12:**
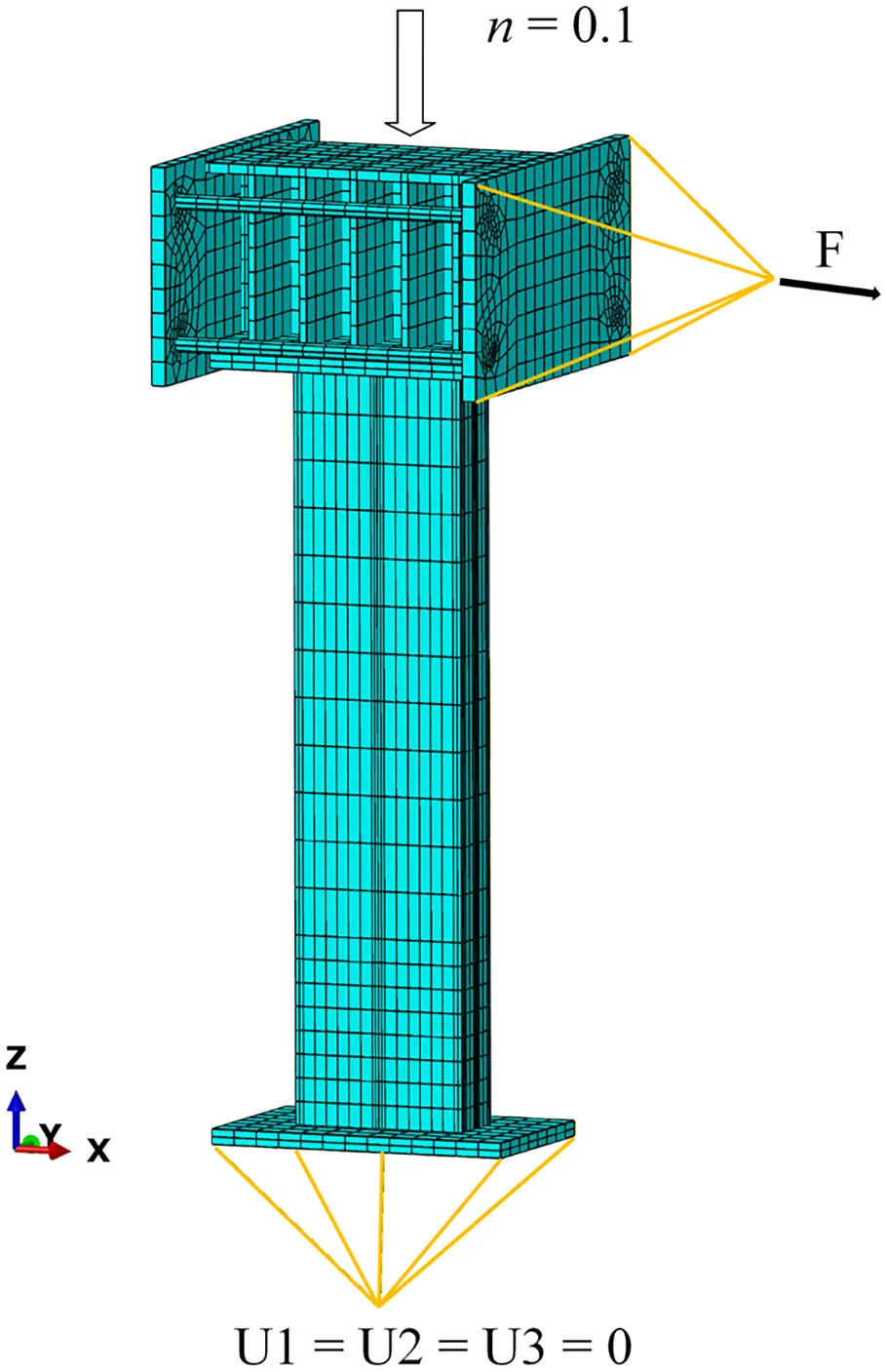
Finite element model for cyclic analysis.

The concrete material properties in the cyclic analysis were the same as those in the axial-compression analysis, and the CDP model was still employed.

Under repeated loading, concrete cracks may open and close, and accumulated irreversible damage develops; thus, a damage factor is typically introduced. Following Ref [17]., the present study combined the energy equivalence and strain equivalence hypotheses, Eq. (12) defines the calculation of the concrete plastic damage factor.


$$\begin{array}{c} d=\text{1}-\sqrt{\text{1}-D} \end{array}$$ (12)

where *d* is the concrete plastic damage factor; *D* is the concrete damage evolution parameter.

For the steel material of the specimens, a combined hardening model was adopted to describe the cyclic elastoplastic response of steel. In the combined hardening model, yield strength and elastic modulus were input based on material test results, and the coefficients controlling kinematic and cyclic hardening were set according to Ref [16].

A reference point RP-1 was defined at the center of the side face of the loading device and coupled with that face. The horizontal low-cycle reversed cyclic load was applied at RP-1. To simulate the vertical load in tests, a corresponding vertical pressure was applied on the top surface of the loading device, and the axial load ratio was set to 0.1. The bottom surface of the base plate was constrained in the X, Y, and Z directions (U1 = U2 = U3 = 0). The loading protocol followed Ref [16]., with drift ratios of 0.375%, 0.5%, 0.75%, 1%, 2%, 3%, and 4%, and one cycle applied at each drift level. The drift-ratio sequence was selected with reference to the experimental loading protocol, while one cycle was applied at each drift level in the FE analysis to improve computational efficiency and ensure consistent comparison among the parametric models. Since the same simplified loading protocol was used for all specimens, the results are mainly used to compare their relative cyclic responses. Because one cycle was applied at each drift level and steel fracture was not explicitly modeled, the cyclic analysis mainly focuses on the comparative lateral load-bearing capacity and hysteretic response of different specimens under the adopted loading protocol.

### 4.2 Simulation results

Fig 13 shows the von Mises equivalent stress contours of the steel tube for the cyclic-analysis specimens. Fig 14 shows the corresponding stress contours of the concrete.

**Fig 13:**
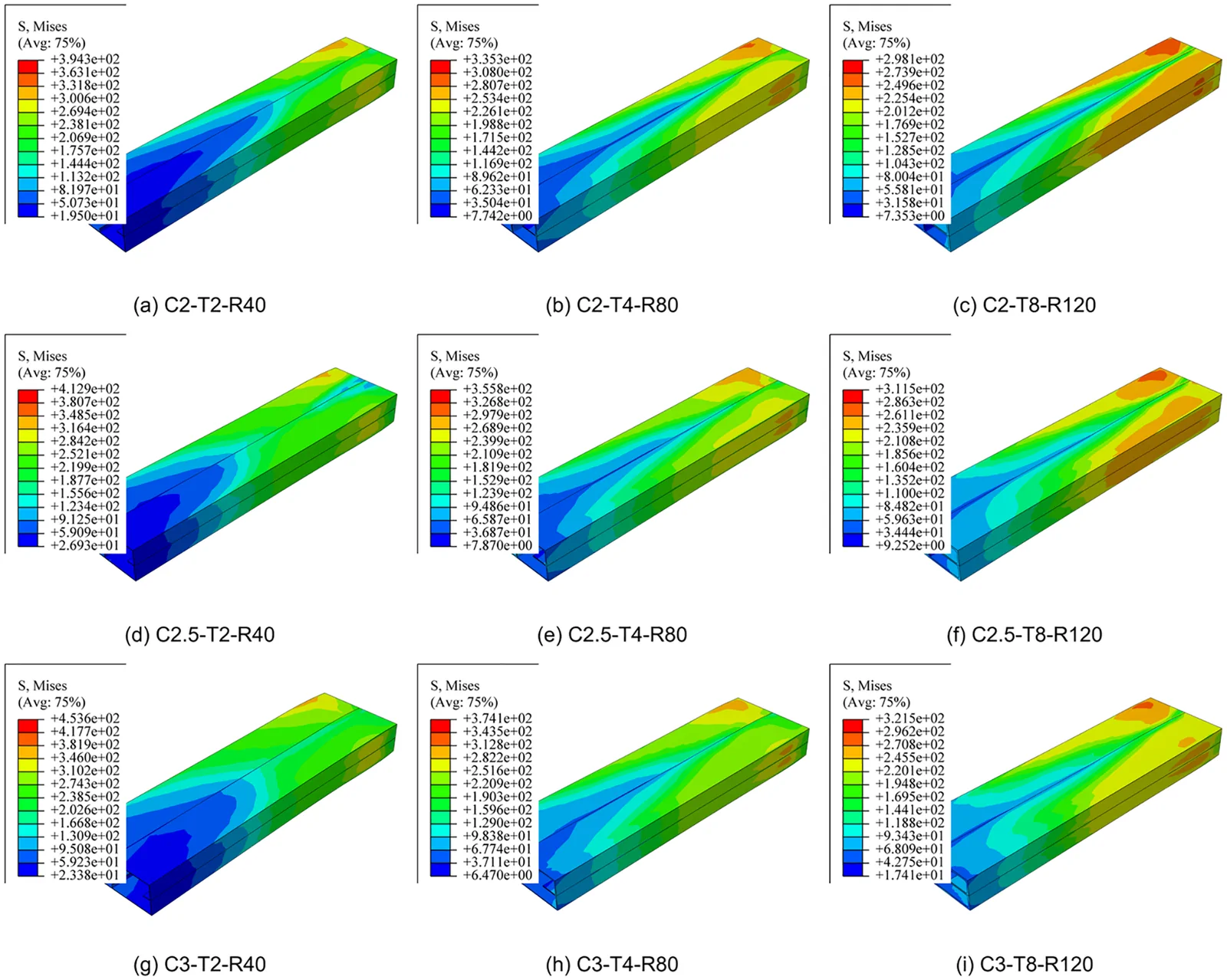
Steel-tube stress distributions of cyclic-analysis specimens.

As shown in Figs 13–14, the maximum stresses of the nine stiffened flat columns concentrate near the bottom region of the tube walls. Comparing specimens with the same aspect ratio, as the wall thickness increases, the steel-tube stress decreases while the internal concrete stress increases. This indicates that thicker steel tubes provide stronger confinement to the core concrete, thereby more effectively sharing the axial load: the concrete carries higher stress while the steel stress is relatively reduced, reflecting improved composite action.

**Fig 14:**
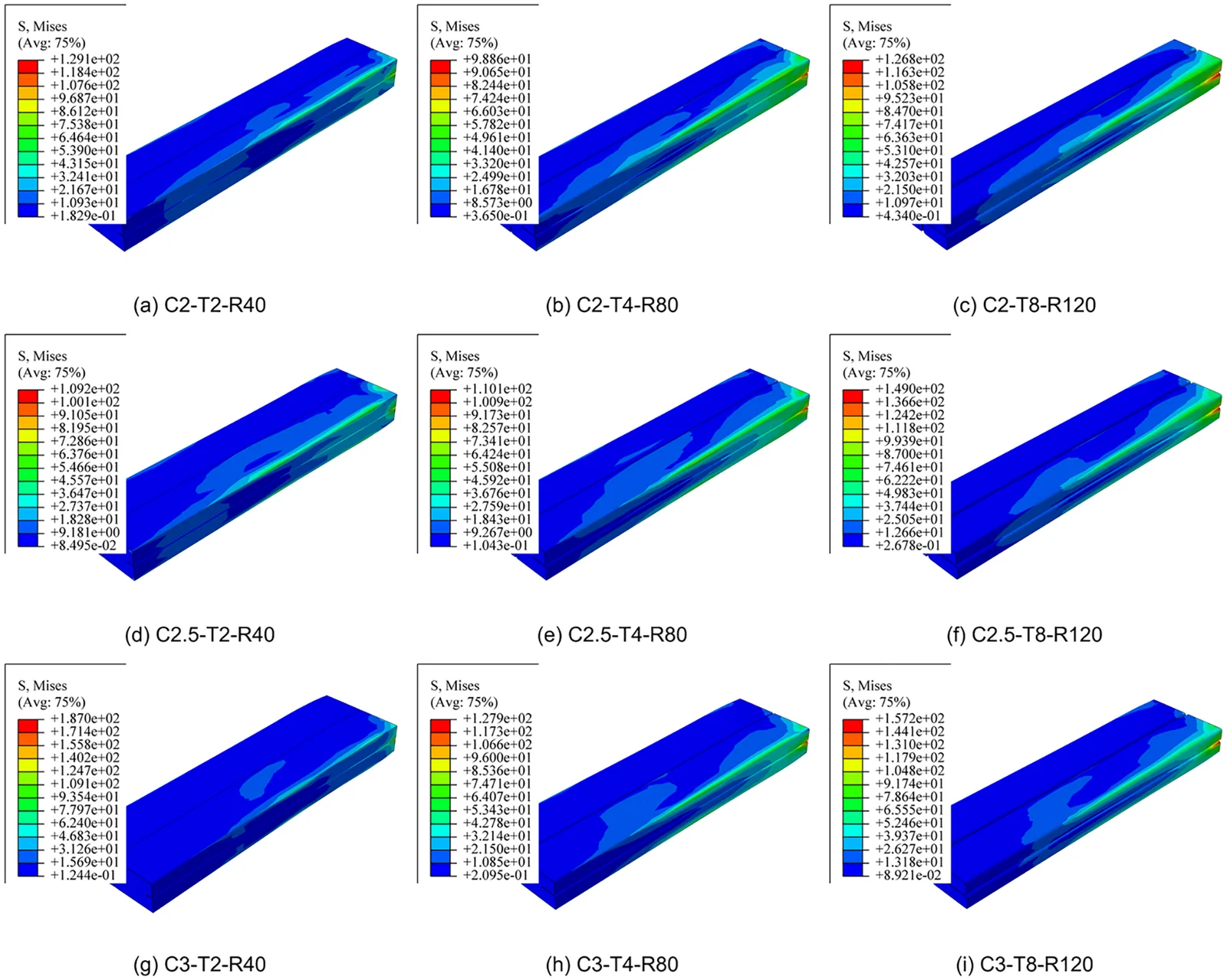
Concrete stress distributions of cyclic-analysis specimens.

Fig 15 shows the hysteresis curves of flat columns with different wall thicknesses. Fig 16 shows the hysteresis curves of flat columns with different aspect ratios.

**Fig 15:**
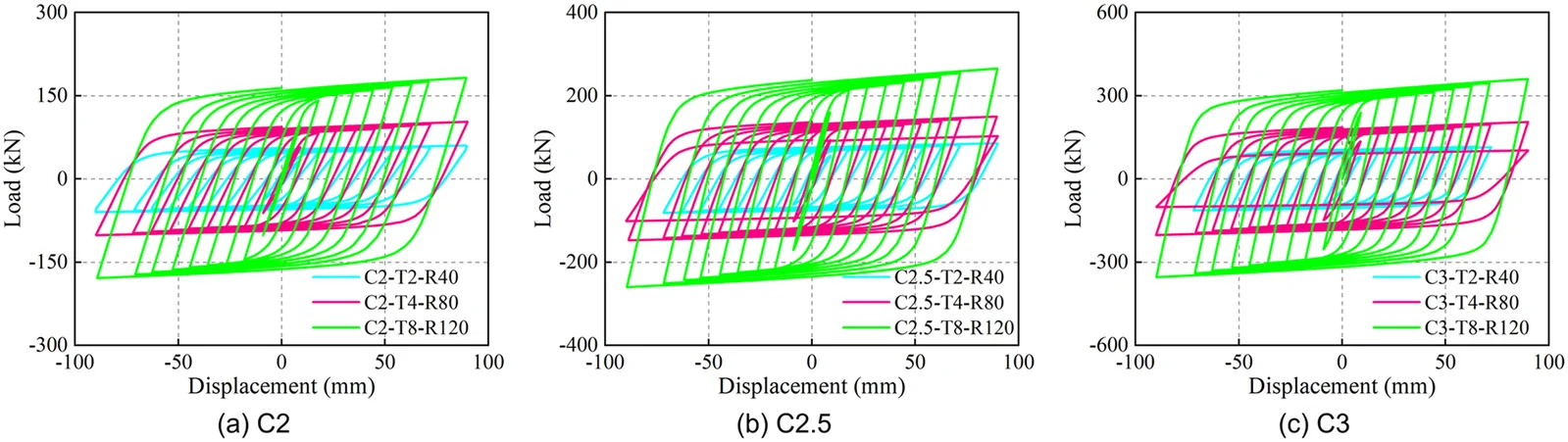
Hysteresis curves of flat columns with different tube thicknesses and corresponding rib dimensions.

**Fig 16:**
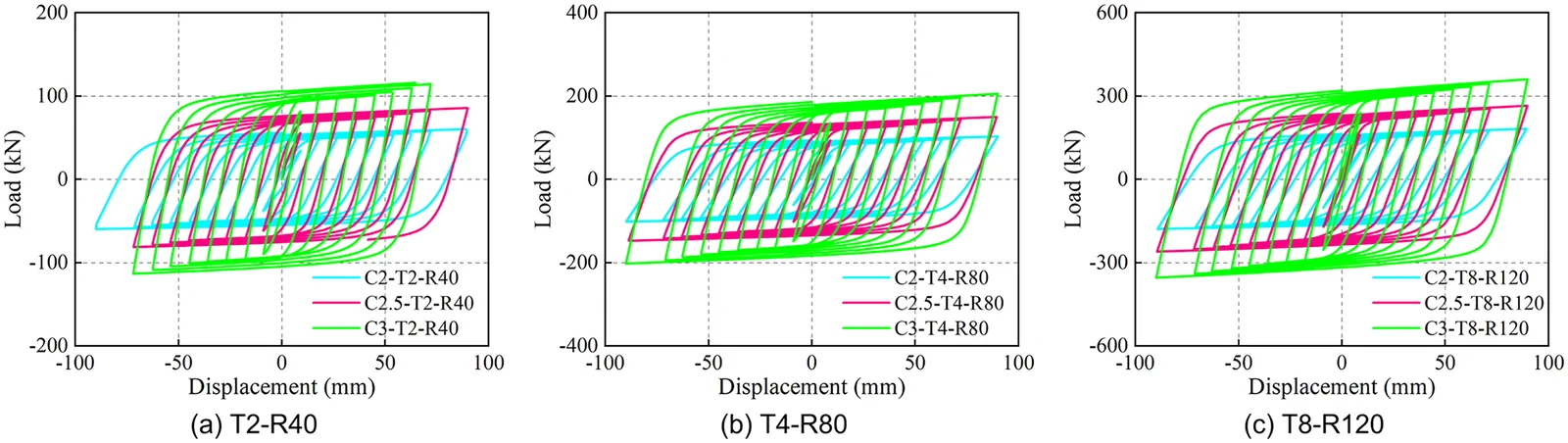
Hysteresis curves of flat columns with different aspect ratios.

Figs 15–16 show that the nine stiffened specimens have full hysteresis loops. This indicates stable cyclic behavior and good energy dissipation under the applied loading protocol. At the same section aspect ratio, the loops become larger from T2-R40 to T4-R80 and T8-R120. The lateral capacity and energy dissipation also increase. In these models, tube thickness and rib dimensions change at the same time. Their separate effects on cyclic behavior cannot be identified. At the same thickness-rib group, increasing the section aspect ratio from 2.0 to 3.0 moderately increases the lateral capacity. This is mainly due to the larger section depth and flexural resistance. Overall, the results show that the coupled strengthening scheme improves the cyclic response within the studied model matrix.

Fig 17 compares the hysteresis curves of the non-stiffened specimen C2-T2-R0 and the stiffened specimen C2-T2-R40. The two specimens have the same outer section size and tube thickness, and the only difference is the presence of internal longitudinal ribs. Compared with C2-T2-R0, the hysteresis loop of C2-T2-R40 is fuller, and the enclosed area is larger. The lateral load capacity increases by 12.2%, indicating that the integrated cold-formed ribs improve the cyclic load-bearing capacity and energy dissipation of the flat column under the adopted loading protocol.

**Fig 17:**
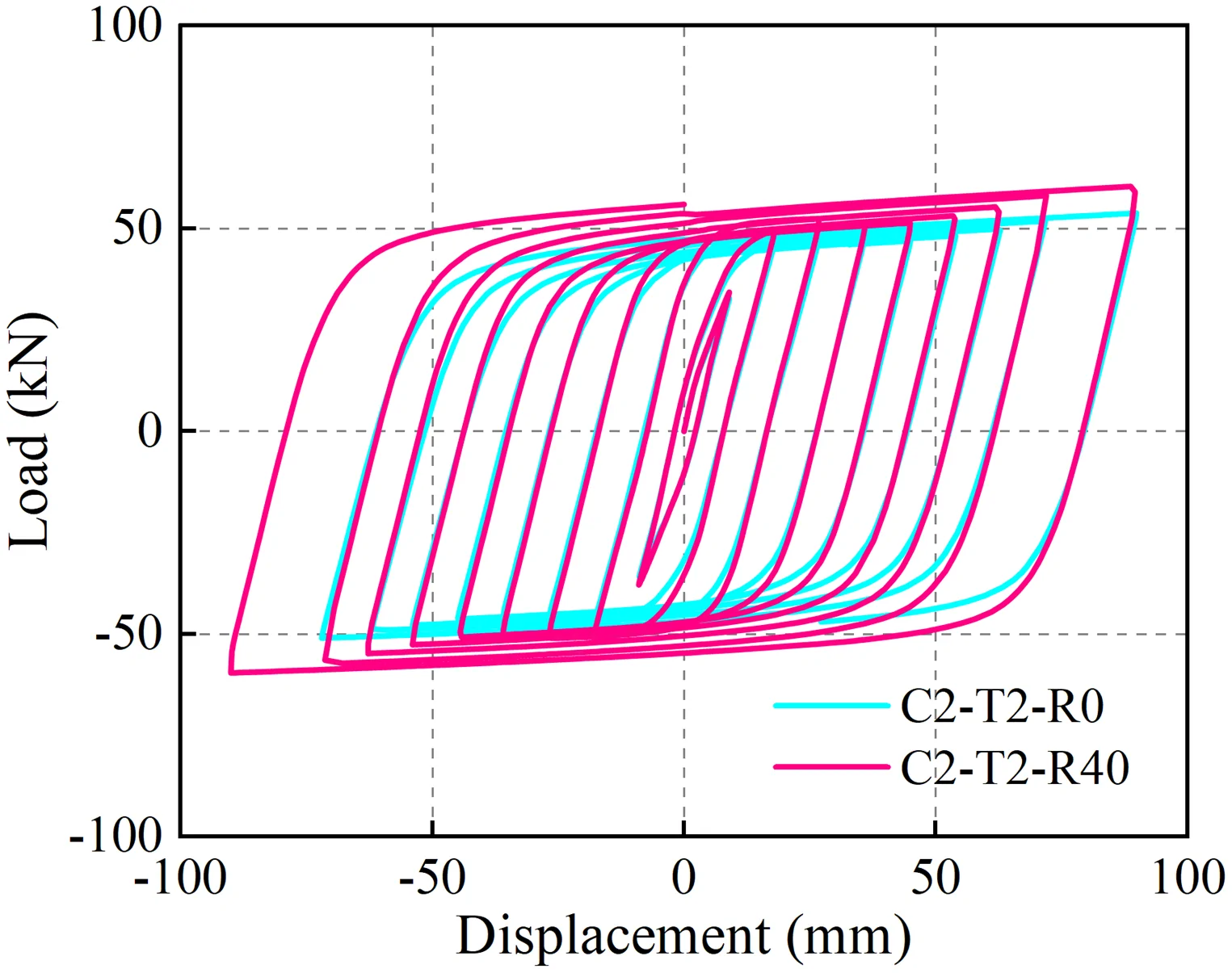
Hysteresis curves of the non-stiffened and stiffened specimens.

## 5. Conclusions

To address insufficient confinement and local outward buckling of conventional CFST flat columns, this study proposed a stiffened cold-formed built-up CFST flat column assembled from four lipped channel plates. Three-dimensional nonlinear FE models were developed in ABAQUS to investigate the axial-compression and quasi-static cyclic behavior. In addition to the original specimen matrix, a supplementary decoupled parametric analysis was conducted to distinguish the individual effects of tube thickness and rib dimensions. The main conclusions are as follows:

(1) The proposed stiffened configuration improves the mechanical behavior of CFST flat columns. Compared with the non-stiffened specimen with the same outer section size and tube thickness, the stiffened specimen shows higher axial and lateral load-bearing capacity, fuller hysteresis loops, and better post-peak or cyclic load retention. The integrated cold-formed ribs provide additional local restraint to the flat steel plates and improve the steel-concrete composite action.
(2) In the original specimen matrix, tube thickness and rib dimensions were increased simultaneously. The coupled strengthening scheme improves the axial-compression and cyclic responses. The improvement is reflected in higher load-bearing capacity, larger hysteresis loops, and increased energy dissipation. However, the two parameters were not varied independently in the cyclic models. Their individual contributions under cyclic loading therefore cannot be determined from the present results.
(3) The supplementary decoupled analysis indicates that tube thickness is the dominant parameter governing axial load-bearing capacity. Increasing tube thickness directly increases the steel area, reduces the plate width-to-thickness ratio, and enhances confinement of the core concrete. In contrast, increasing rib dimensions at a fixed tube thickness produces a smaller increase in peak axial load, but it helps reduce local outward deformation and improve residual load retention.
(4) Within the investigated range, increasing the section aspect ratio from 2.0 to 3.0 increases the axial and lateral load capacity. This trend is mainly associated with the increase in section size, section depth, and flexural resistance. However, because the present study is based on FE analysis and the cyclic loading protocol applies one cycle at each drift level, further experimental studies and multi-cycle loading analyses are needed to directly validate the proposed configuration and evaluate cumulative damage effects.

## Supporting information

S1 Data Raw numerical data. (XLSX)
